# Beneficial Modulatory Effects of Treatment With Bone Marrow Lysate on Hematopoietic Stem Cells and Myeloid Cells in Tumor-Bearing Mice

**DOI:** 10.3389/bjbs.2022.10328

**Published:** 2022-06-29

**Authors:** Mohamed L. Salem, Kadry A. El-Bakry, Eman H. Moubark, Ashraf Sobh, Sohaila M. Khalil

**Affiliations:** ^1^ Immunology and Biotechnology Unit, Zoology Department, Faculty of Science, Tanta University, Tanta, Egypt; ^2^ Center of Excellence in Cancer Research (CECR), Tanta University, Tanta, Egypt; ^3^ Zoology Department, Faculty of Science, Damietta University, Damietta, Egypt; ^4^ Department of Biology, Faculty of Science, Jazan University, Jazan, Saudi Arabia

**Keywords:** bone marrow, stem cells, CTX, G-CSF, myeloid derived suppressor cells, leukopenia, lysate

## Abstract

**Introduction:** Leukopenia is one of the major side effects of myelosuppressive chemotherapy such as cyclophosphamide (CTX). We and others have used CTX either alone or in combination with G-CSF for the mobilization of hematopoietic stem cells (HSCs). This mobilization can induce expansion of myeloid cells with immunosuppressive phenotype. In this pilot study, we aimed to test whether bone marrow lysate (BML)/CTX, a rich source of growth factors, can lower the expansion of myeloid cells with immunosuppressive phenotypes in tumor-bearing mice without interfering with the anti-tumor effects of CTX or with the mobilization of HSCs.

**Methods:** Female CD1 mice were treated on day 0 with an i.p. injection of Ehrlich ascites carcinoma (EAC). On day 7, the mice were i.p. injected with CTX followed by s.c. injection of G-CSF for 5 consecutive days, single s.c. injection of BML/PBS or BML/CTX or single i.v. injection of BMC/PBS or BMC/CTX.

**Results:** Treatment of EAC-bearing mice with BML/PBS or BML/CTX did not interfere with the anti-tumor effect of CTX. EAC increased the numbers of immature polymorphonuclear cells (iPMN; neutrophils) in both blood and spleen. Treatment of EAC-bearing mice with CTX further increased the numbers of these cells, which were decreased upon treatment with BML/CTX. Treatment with BML/PBS or BML/CTX increased the numbers of stem cells (C.Kit^+^Sca^-^1^+^) in BM; the effect of BML/CTX was higher, but with no significant effect on the numbers of HSCs. Future studies are needed to analyze the molecular components in BM lysate and to determine the underlying mechanisms.

## Introduction

Cyclophosphamide (CTX) is a common chemotherapeutic agent that is used clinically for the treatment of several human malignancies [1]. We have shown in a series of previous preclinical studies that the application of a lymphodepletion regimen prior to the adoptive transfer of T cells can significantly improve the responses of the transferred T cells including their activation, proliferation, and functions. Furthermore, these responses were associated with marked enhancement of the anti-tumor immunity of these cells [2]. Despite this beneficial effect of CTX for adoptive T cell therapy, leukopenia is the major side effect in cancer patients who receive CTX and other myelosuppressive chemotherapy [3, 4]. As such, several studies have focussed on finding approaches to lower leukopenia without eliminating the anti-tumor effects of CTX as well as its cancer immunotherapy benefits.

The most effective approach to reducing CTX-induced leukopenia is the use of G-CSF for the mobilization of hematopoietic stem cells (HSCs) from bone marrow (BM) to the circulation [5]. This mobilization increases the numbers of circulating progenitor cells and stimulates granulopoiesis, ending up in increased neutrophil numbers. This process often shortens the period of severe leukopenia after high-dose chemotherapy and BM transplantation [6].

A fine balance between self-renewal and the production of the differentiated progeny of stem and progenitor cells is necessary to generate cellular diversity during development and maintain adult tissue homeostasis [7]. Disruption of this balance may result in premature depletion of the stem/progenitor cell pool, or abnormal growth. This would explain why mobilization of HSCs is often associated with the emergence of cells expressing the phenotype of myeloid-derived suppressive cells (MDSCs) [8, 9], which play a pivotal role in tumor progression in mice and cancer patients [10].

MDSCs represent an intrinsic part of the myeloid-cell lineage. They are a heterogeneous population that is comprised of myeloid-cell progenitors and precursors of myeloid cells [11]. They expand as immature cells in the BM or even extramedullary (mainly in the spleen) and migrate into the peripheral blood where their terminal differentiation is blocked and then is transformed finally into functionally active MDSCs [12]. A significant correlation between circulating MDSC and clinical cancer stage was observed [13]. Once the production of MDSC-prone cytokines and chemokines are blocked, the formation of MDSCs decreases [14]. As the enrichment and activation of MDSCs seem to be a general characteristic of malignant diseases, targeting these cells could be applied to treat various tumor entities [15, 16].

Several studies have shown that different hematopoietic cells, including BM cells [17], platelets [18], and peripheral blood leukocytes [19] are rich in growth factors and cytokines. These factors have a great beneficial therapeutic potential in different disease settings. These studies would indicate that BM cell lysates may induce stem cell mobilization and the release of myeloid cells in particular in a leukopenic host we have found that unfractionated peripheral blood mononuclear cells (PBMCs) harvested from the peripheral blood of patients with leg ischemia who were pretreated with G-CSF-based mobilizing protocol showed accelerated wound healing [20]. We further found that these clinical beneficial effects were associated with a high number of W.B.Cs, and HSCs in the peripheral blood (PBL) of ischemic patients after 5 consecutive injections of G-CSF. This reported effect was attributed to the infused cells’ paracrine production of growth factors [20]. According to prior reports from clinical investigations, the therapeutic capacity of BMC on the peripheral blood leucocytes to treat acute myocardial infraction was associated with the release of paracrine growth factors and cytokine mediators [21, 22].

Given the above-mentioned data indicating that BM is a potential source of growth factor with beneficial effects, we designed this pilot study to test whether BM lysates can lower the expansion of myeloid cells with immunosuppressive phenotypes in tumor bearing mice treated with CTX without interfering with the expansion of stem cells or with the anti-tumor effects of CTX.

## Materials and Methods

### Mice

Female CD1mice (8-weeks old), weighing 20–25 gm were purchased from the Faculty of Pharmacy, Mansoura University, Egypt, and used in the study (there was a total number of 6 mice in each group). Mice were housed at the Animal House, Zoology Department, Faculty of Science, Tanta University in accordance with the internal guidelines for the use of experimental animals. The research study was approved by the Ethical Committee, Faculty of Science, Tanta University, Egypt, before the commencement of the study (REC-FSTU). All methods were carried out in accordance with relevant guidelines and regulations and under the approval of IRB (IACUC-SCI-TU-0271).

### Reagents and Cell Line

CTX commercially known as Endoxan^®^ was purchased from Aldrich-Sigma Company, United States and reconstituted in 50ml PBS as a stock solution, and kept at −80°C until use. G-CSF, commercially known as Neupogen, was purchased from Biopharmaceutical Co.; Ltd., Egypt. Ehrlich ascites carcinoma (EAC) cell line was purchased from the National Cancer Institute, Cairo University, Egypt. The tumor cell line was maintained in CD1 female albino mice by serial intraperitoneal (i.p.) transplantation of 2.5 x l0^5^ cells/mouse suspended in 0.1 ml PBS sterile.

### Monoclonal Antibodies

Rat anti-mouse CD11b-APC labeled, Ly6G- FITC labeled, C. KIT-FITC labeled, and Sca-1- PE labeled monoclonal antibodies were purchased from BD (Pharmingen, San Diego, CA, United States). They were used for flow cytometric analysis according to manufacturer instructions.

### Tumor Challenge, Chemotherapy, and Bone Marrow Treatment

Mice were divided into seven groups; each group included 6 mice. All groups were injected i.p with 2.5 × 10^5^ cells of viable EAC. After 7 days mice were injected i.p with PBS or CTX (200 mg/kg b.wt). The next day after CTX injection, mice were treated with G-CSF (250 μg/kg/d), single s.c. injection with BM lysate (BML; 100 μg/mouse/d) prepared from naïve (BML/PBS) or CTX-treated (BML/CTX) mice, respectively for five consecutive days. Single intravenous (i.v.) injection of BM cells (5 × 10^6^ cells) harvested from naïve (BMC) or CTX (BMC/CTX) treated mice. One day after the last injection, blood samples were collected using orbital bleeding then mice were sacrificed to obtain tumor cells, bone marrow, and splenocytes.

### Preparation of Bone Marrow Cells and Lysate

Briefly, BM cells were collected and washed twice in PBS and then re-suspended at a density of 5 × 10^6^ cells/ml in PBS. The cell suspension was disrupted by four freeze-thaw cycles. The cell lysate was centrifuged for 10 min at 300 x g to remove crude debris. The supernatant was collected and passed through a 0.2 µm filter. Protein concentration was determined by a commercial Bio-Rad protein assay and aliquots were frozen at 80°C until use.

### Flow Cytometry

Erythrocytes were depleted in blood and spleen with ACK buffer (Invitrogen, Carlsbad, CA)**.** Spleen cell suspensions were prepared and counted using a hemocytometer with trypan blue dye exclusion. To differentiate the myeloid cell subsets, cells were stained with mAbs against CD11b (FITC) and Ly6G (APC) myeloid. To identify the different subsets of HSCs in BM, cells were stained with mAbs C. Kit (FITC) and Sca-1[8]. The cells were then washed twice with PBS and then acquired using a Partec flow cytometer and analyzed using flow Jo software (BD Biosciences). The HSCs were characterized as C-Kit + Sca-1+ and myeloid cells were characterized as Ly6G^+^CD11b^+^ (Immature neutrophils; iPMN), Ly6G^−^CD11b^+^ (monocytes in blood and macrophages in spleen), and Ly6G^+^CD11b^−^ (mature neutrophils; mPMN).

### Statistical Analysis

Numerical data obtained from each experiment were expressed as mean ± SE and the statistical differences between experimental and control groups were assessed using One-way Analysis of Variance (ANOVA). GraphPad Prism (GraphPad Software, Inc., San Diego, CA) was used to analyze *p* values. *p* values ≤0.05 were considered statistically significant.

## Results

### Treatment With Bone Marrow Products Does Not Interfere With the Anti-Tumor Effect of Cyclophosphamide

**FIGURE 1:**
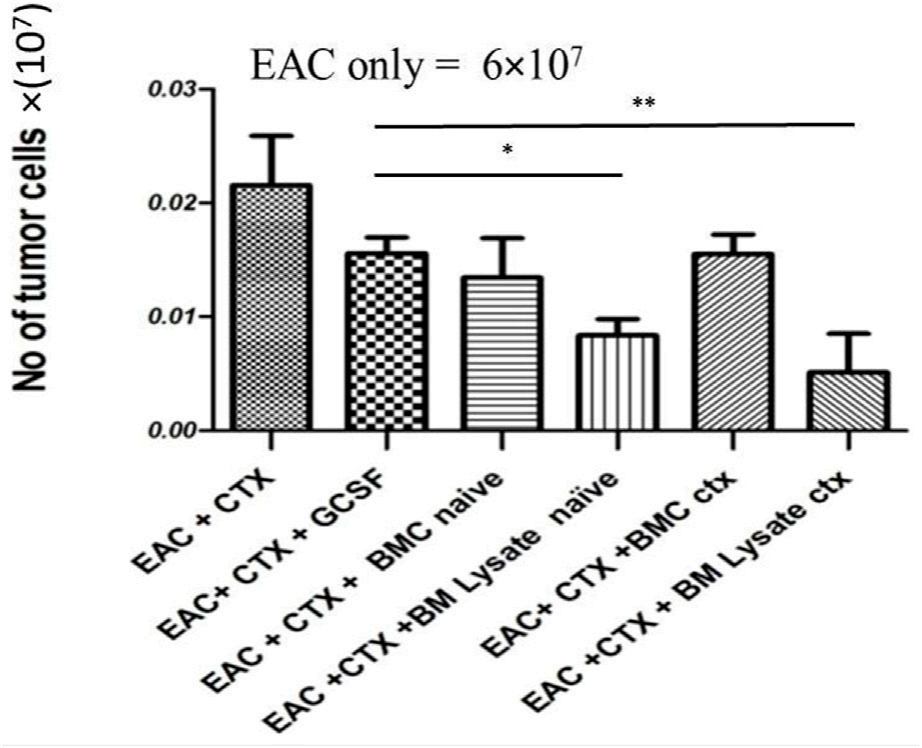
BM products do not interfere with the anti-tumor effects of CTX in experimental groups: Mice were injected with EAC. The next day they were treated with either PBS or CTX or G-CSF, BM lysate prepared from naïve donor mice, CTX-treated donor mice, or BM cells harvested from naïve donor mice, and CTX-treated donor mice. Then mice were sacrificed and ascites collected on day 7. * Refers to *p* = 0.05. ** Refers to *p* ≤ 0.05 Columns represent the mean ± SE (*n* = 6). Abbreviations; EAC, Ehrlisch Ascites carcinoma; CTX, cyclophosphamide; G-CSF, granulocyte-colony stimulating factor; BMC naive, bone marrow cells extracted from Naïve mice; BMC CTX, bone marrow cells extracted from CTX-treated mice; BML naive, bone marrow lysate extracted from Naïve mice; BML CTX, bone marrow lysate extracted from CTX-treated mice.

As expected, CTX treatment induced a potent antitumor effect. The tumor cell count after treatment with CTX and either with BML/PBS (*p* value = 0.0279) or BML/CTX (*p* value = 0.0246) further enhanced the anti-tumor effects. Treatment with BMC/PBS or BMC/CTX or G-CSF without CTX, however, showed no significant decreases in tumor cell count, as compared to EAC/CTX in (Figure 1).

### Treatment With BML/CTX Decreased the Numbers of Myeloid Cells in Peripheral Blood and Spleen

**FIGURE 2:**
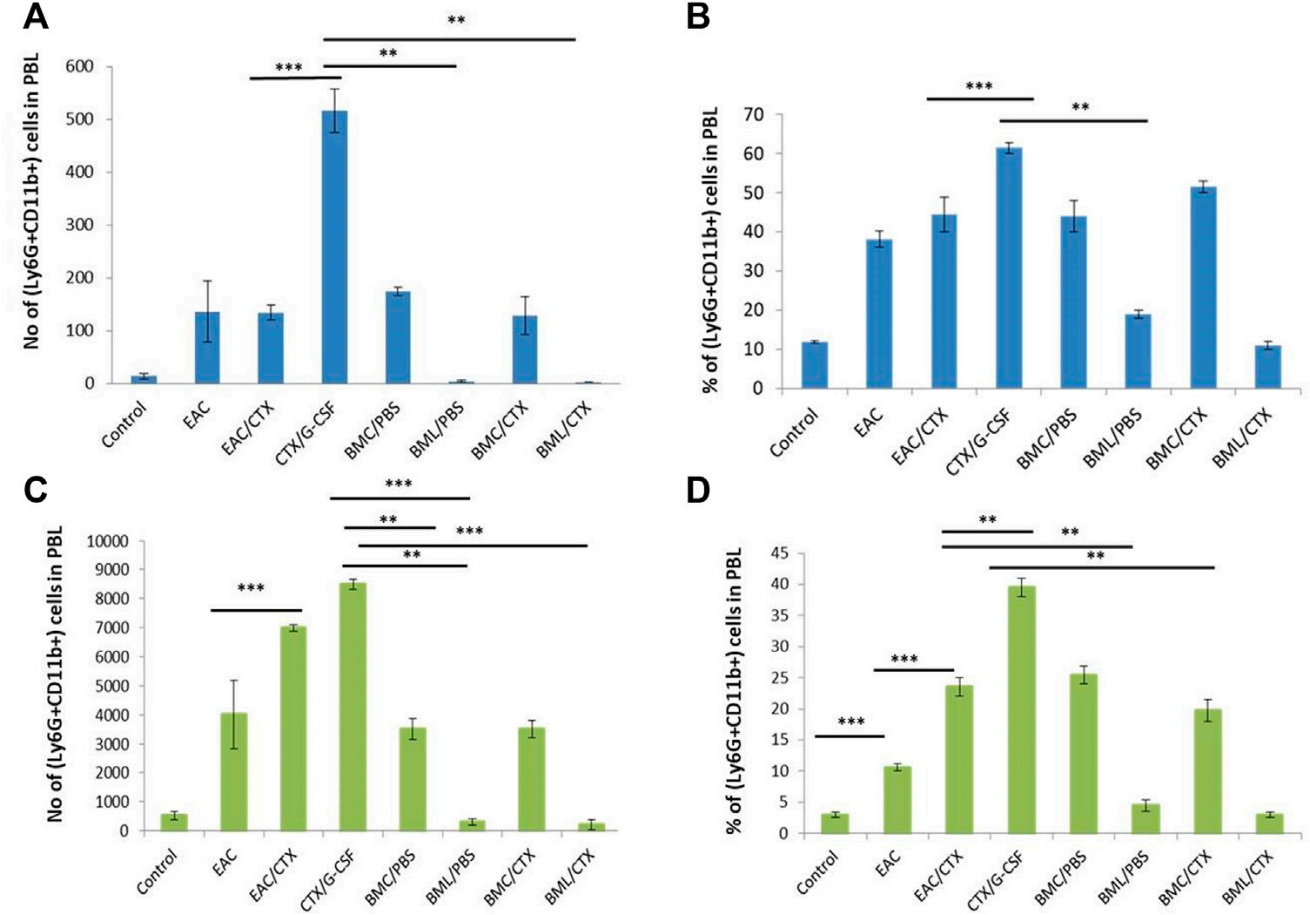
The total number and the relative number of myeloid cell subpopulations of immature neutrophils in PBL and spleen. **(A)** Total number of Ly6G+CD11b+ in PBL. **(B)** The percentage of Ly6G+CD11b+ in PBL. **(C)** Total number of Ly6G+CD11b+ in the spleen. **(D)** The percentage of Ly6G+CD11b+ in spleen. *** Refers to *p* < 0.001. Columns represent the mean ± SE (*n* = 6). Abbreviations: EAC, Ehrlisch Ascites carcinoma; CTX, cyclophosphamide; G-CSF, granulocyte-colony stimulating factor; BMC/PBS, bone marrow cells extracted from Naïve mice; BMC/CTX, bone marrow cells extracted from CTX-treated mice; BML/PBS, bone marrow lysate extracted from Naïve mice; BML/CTX, bone marrow lysate extracted from CTX-treated mice.

**FIGURE 3:**
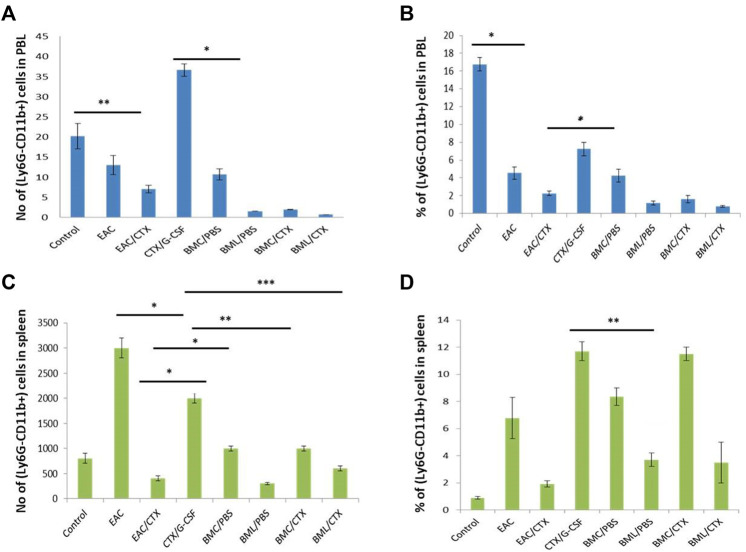
The total number and the relative number of myeloid cell subpopulations of monocytes in the PBL and macrophage of the spleen. **(A)** total number of Ly6G-CD11b+ in PBL **(B)** The percentage of Ly6G-CD11b+ in PBL. **(C)** Total number of Ly6G-CD11b+ in spleen **(D)** The percentage of Ly6G-CD11b+ in spleen. Columns represent the mean ± SE (*n* = 6). * Refers to *p* = 0.05, ** Refers to *p* ≤ 0.05, *** Refers to *p* < 0.001. Abbreviations: EAC, Ehrlisch Ascites carcinoma; CTX, cyclophosphamide; G-CSF, granulocyte-colony stimulating factor; BMC/PBS, bone marrow cells extracted from Naïve mice; BMC/CTX, bone marrow cells extracted from CTX-treated mice; BML/PBS, bone marrow lysate extracted from Naïve mice; BML/CTX, bone marrow lysate extracted from CTX-treated mice.

Supplementary Figure S1 shows the gating strategy of myeloid cells in the PBL; including immature neutrophils (Ly6G+CD11b+), mature neutrophils (Ly6G+CD11b-), and monocytes in the spleen macrophages (Ly6G-CD11b+). The data in Figure 2 reveal that EAC-bearing mice showed significant increases in the relative and absolute numbers of iPMN in PBL (Figures 3A,B; *p* value = 0.12) and splenocytes (by 3.6- fold) as compared to control (Figures 3C,D). Treatment of EAC-bearing mice with CTX resulted in no change in the relative and the absolute numbers of iPMN in the PBL (Figures 2A,B) as compared to EAC-bearing mice and showed an increase in splenocytes (Figures 2C,D), *p* value ≤0.05 by 1.2 and 2.5- fold, respectively, as compared to EAC-bearing mice. Treatment with CTX/G-CSF induced further increases in the relative and the absolute number of iPMN by 2.5- 1.5-fold as compared to EAC/CTX (*p* = 0.001). Treatment with BMC/PBS or BMC/CTX after treatment with CTX induced increases in the relative and absolute numbers of iPMN by about 1.5-fold in the PBL (Figures 2A,B) as compared to CTX/G-CSF (*p* = 0.32). In the spleen, however, treatment with BMC/PBS or BMC/CTX (*p* = 0.01) showed decreases by 1.5-fold in the relative and absolute numbers of iPMN as compared to CTX/G-CSF (Figures 2C,D). Interestingly, treatment with BML/PBS or BML/CTX showed significant decreases in the relative and absolute numbers of iPMN by 3- folds (*p* = 0.0223) in the PBL (Figures 2A,B) and about 8-fold in the spleen as compared to CTX/G-CSF (Figures 2C,D).

### Treatment With BML/CTX Showed Decreased Number of Mature Neutrophils in the Peripheral Blood and Spleen

The data in Supplementary Figure S2 illustrate that EAC-bearing mice showed significant increases in the relative and absolute numbers of mPMN in PBL (Supplementary Figures S2A,B) and spleen in (Supplementary Figures S2C,D) by 3 and 7-fold, respectively, as compared to the control (*p* = 0.001). Treatment of EAC-bearing mice with CTX/G-CSF did not alter the relative and the absolute numbers of mPMN (*p* = 0.21 in the PBL and spleen as compared to EAC-bearing mice. Treatment with CTX/G-CSF, however, showed increases in both the relative and absolute numbers of these cells by 3.5 and 2.5-fold in PBL (Supplementary Figures S2A,B) and spleen (Supplementary Figures S2C,D), respectively as compared to EAC/CTX (*p* = 0.01). Although, treatment with BMC/PBS or BMC/CTX (*p* = 0.001) showed a decrease in the relative and absolute numbers of mPMN as compared to CTX/G-CSF in the PBL (Supplementary Figures S2A,B) by 4-fold it showed a 2.3- fold decreases in the relative and absolute numbers of these cells in the spleen (Supplementary Figures S2C,D) as compared toCTX/G-CSF. Treatment with BML/PBS or BML/CTX (*p* = 0.029) showed significant decreases in the relative and absolute numbers of mPMN by about 12.5- 8- fold in the PBL (Supplementary Figures S2A,B), and spleen (Supplementary Figures S2C,D), respectively as compared to CTX/G-CSF.

### Treatment With BML/CTX Showed Decreased Number of Monocytes in the Peripheral Blood and Macrophages in the Spleen

EAC- bearing mice showed significant decreases (5-fold) in the relative and absolute numbers of monocytes in PBL as compared to the control However, the spleens in these mice showed increases (8-fold) in the relative and absolute numbers of macrophages as compared to control mice. Treatment of these mice with CTX induced decreases in the relative and the absolute numbers of monocytes and macrophages by 3.8- and 5-fold, respectively, in PBL and spleen as compared to EAC-bearing mice. Treatment with both CTX and G-CSF induced increases (*p* = 0.01) in the relative number of monocytes and macrophages 1.5- and 3-fold in PBL (Figures 3A,B) and spleen (Figures 3C,D), respectively as compared to EAC/CTX. Treatment with BMC/PBS or BMC/CTX after CTX/G-CSF did not have further effects. However, treatment with BML/PBS or BML/CTX after CTX/G-CSF treatment showed significant decreases (*p* = 0.012) in the relative and the absolute number of monocytes and macrophages by 2.7- and 3.5- old, respectively, in PBL (Figures 3A,B) and spleen in (Figures 3C,D) as compared to CTX/G-CSF.

### Treatment With BML/CTX Showed an Increase in the Numbers of Hematopoietic Stem Cells in Bone Marrow

**FIGURE 4:**
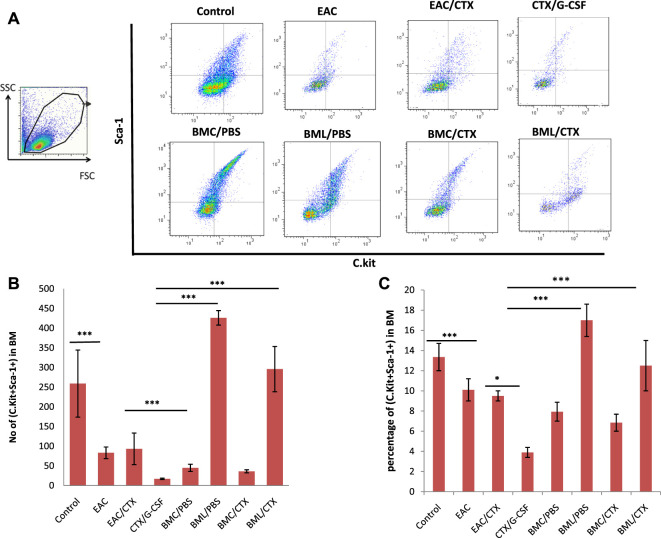
Representative data of Flow cytometry analysis: **(A)** to illustrate the gating strategy of hematopoietic stem cells subpopulations in BM. They include **C**. kit + Sca-1+ hematopoietic stem cells, C.Kit- Sca-1+ adult hematopoietic stem cells, and C.Kit + Sca-1- common myeloid progenitor in BM. **(B)** Total number of C.Kit + Sca-1+ adult hematopoietic stem cells **(C)** The percentage of C.Kit + Sca-1+ adult hematopoietic stem cells. Columns represent the mean ± SE (*n* = 6). *** Refers to *p* < 0.001. Abbreviations: EAC, Ehrlisch Ascites carcinoma; CTX, cyclophosphamide; G-CSF, granulocyte-colony stimulating factor; BMC/PBS, bone marrow cells extracted from Naïve mice; BMC/CTX, bone marrow cells extracted from CTX-treated mice; BML/PBS, bone marrow lysate extracted from Naïve mice; BML/CTX, bone marrow lysate extracted from CTX-treated mice.

On day 7 post EAC challenge, mice were sacrificed and BM was collected and analyzed by flow cytometry to evaluate the effect of the treatment with BM products following CTX treatment on BM HSCs (C-kit^+^Sca-1^+^). The gating strategy of HSCs is shown in Figure 4A. EAC-bearing mice showed significant decreases (4-fold) in the relative and absolute numbers of HSCs as compared to control (*p* ≤ 0.001). Of note, CTX treatment did not alter the effect of the tumor on the number of HSCs (Figures 4B,C). Treatment with both CTX and G-CSF induced a higher decrease in the relative and absolute numbers of HSCs in EAC-bearing mice (1.5-fold) (*p* = 0.002). Treatment with BMC/PBS or BMC/CTX after preconditioning with CTX showed increases in the relative and total numbers of HSCs by 2.7 and 1.2- folds, respectively as compared to treatment with CTX/G-CSF (*p* ≤ 0.001). Treatment with BML/PBS or BML/CTX showed significant increases (3.5-fold) in the relative and absolute numbers of HSCs as compared to treatment with CTX/G-CSF (Figures 4B,C) (*p* ≤ 0.001).

## Discussion

Leukopenia is one of the major side effects of myelosuppressive chemotherapy such as CTX [23]. Recovery from leukopenia can be overcome by treatment with G-CSF, which acts through the mobilization of myeloid cell precursors into the bloodstream [24]. However, combinational treatment with G-CSF and CTX is often associated with the emergence of cells expressing the phenotype of MDSCs [9]. Given that BMC has been found as a rich source of growth factors and cytokines [25, 26], our aim in this study was to test the concept that BM lysate can act as a mobilizing factor, at least in part, by mimicking the mobilizing effects of G-CSF in a leukopenic microenvironment. To address this aim, we compared the effects of BML (as a cell-free source), BMC (as a cellular source), and G-CSF (as a molecular source) on the expansion of myeloid cells in tumor-bearing mice, and whether it interferes with the anti-tumor effects of CTX and its mobilizing effect of HSCs. The rationale behind our aim is based on the premise that different growth factors are present in BML which can compensate for the effect of G-CSF.

To study the kinetics of leukopenia and the kinetics of its recovery, we have used CTX-induced leukopenia as an effective animal model [27]. To investigate the impact of BML on the recovery of leukopenia, we used BMC and BML from tumor-bearing mice either treated with PBS or treated 3 days previously with CTX. Indeed, we and others have found at this precise time point (day 3) that BM is rich in myeloid cell precursors [28]. Under this setting BML from CTX-treated mice is expected to have more growth factors than BML of PBS-treated mice. We found in the present study that a combination of CTX with BMC or BML induces higher anti-tumor effects than those of CTX alone. These results are in line with recent findings showing that chemotherapy with exogenous BM followed by vaccination as immunotherapy can provide higher anti-tumor effects than chemotherapy alone [29]. These anti-tumor effects could be attributed to certain factors released by BMC or BML, and its induction of direct and/or indirect anti-tumor effects. These data reveal, at least in part, that BML (BM proteins) do not interfere with the anti-tumor effects of CTX against Ehrlich ascites carcinoma, the model we used in this study. Indeed, we have found before that the success of treatment with BMCs in patients with leg ischemia is due to the release of growth factors by BMCs [20]. These effects resulted in the growth of new vessels developed by the proliferation of endothelial cells in vascular extremities as well as by BM-mobilized HSCs, which are transformed into endothelial progenitor cells. Furthermore, we found that administration of BMC to mice suffering from myocardial infarction [22], suffering from arthritis [30], or colitis, ameliorated the inflammatory responses and increased mice survival. Similar results have been reported after treatment with either mononuclear BM cells or *in vitro* differentiated mesenchymal stem cells [20].

By testing the effects of BM products on CTX-induced leukopenia, we found that BML induced better effects than BMC against leukopenia, where BML from CTX-treated mice was greater than BML from PBS treated mice. The effects of BML/CTX were, at least in part, similar to those of G-CSF/CTX. Although the mechanisms behind the beneficial effects of BML/CTX are not clear, it could be attributed to the presence of various bioactive molecules such as growth factors, cytokines, and chemokines, which can stimulate the mobilization of myeloid cells from BM and its release into the peripheral pool [31]. In this regard, BML, as paste or broth (named a “superfood”) has been used for decades as a food supplement to enhance immunity due to its gelatin, nutrients, amino acid, and mineral content [32].

In the present study, we found that mice treated with BML/CTX showed a 1.5-fold increase in the total numbers of macrophages in their spleens despite the decrease in the total numbers of iPMN and mPMN in PBL and spleen. The beneficial effects of BML are also supported by stimulating effects of BMC from leukopenic animals by influencing the homeostasis of the hematopoietic compartment through transient lymphodepletion followed by rebound replenishment of immune cells pools [33]. This could explain our results of better mobilization of myeloid cells by BML/CTX than BML/PBS. The higher effects of BML than BMCs can also be attributed to the observation that most administered BM cells may be trapped in the lung and cleared, and only a few injected BMCs are left behind [34]. In addition, these differences between BML/CTX and BML/PBS could be because BM from leukopenic hosts possesses higher numbers of stem cells than those in PBS because of the mobilizing capability of CTX itself [35].

The secretome, or the wide arsenal of bioactive chemicals produced by mesenchymal stem cells, has shown significant therapeutic effect in regenerative medicine [36]. Researchers have been able to recreate the anti-inflammatory, angiogenic, and trophic benefits of stem cells using the secretome as an experimental treatment without the need to use the cells themselves. Several reports have suggested that the beneficial effects of stem-based cell therapy are due to paracrine effects such as growth factors or cytokines from the cells, and not due to the differentiation ability of the stem cells themselves [37]. In this regard, several studies have shown that different hematopoietic cells, including BM cells [17, 25], platelets [18], and peripheral blood leukocytes [19] are rich in growth factors and cytokines, in particular insulin-like growth factor 1 (IGF-1) and vascular endothelial growth factor (VEGF). Given that these factors are of great beneficial therapeutic potential in different disease settings, the above-mentioned studies would indicate that BML may induce stem cell mobilization and the release of myeloid cells in particular in the leukopenic microenvironment [38]. Furthermore, the present study showed that a combination of BML/PBS or BML/CTX induced increases in the total numbers of HSCs by 11.4-fold. In numerous types of malignancies, different subsets of human MDSCs have been identified, and it appears that all MDSC phenotypes can be classified into one of three primary subsets, each of which contains multiple cell populations. Monocytic MDSCs (M-MDSCs) have the same morphology as polymorphonuclear MDSCs (PMN-MDSCs), but they have the opposite expression of myeloid markers, whilst more immature MDSCs (early-stage, e-MDSCs) do not. [39]. Our and other groups reported increased levels of MDSCs in patients as compared with healthy controls. The levels of MDSCs continued to increase markedly following the initiation of chemotherapy [40, 41]. We found in the present study that treatment with BML/CTX had a greater effect than BMC/CTX in the numbers of myeloid cells (iPMN and mPMN) as compared to CTX/G-CSF. Both BM products, however, induced increases in numbers of HSCs, where the effect of BML/CTX was better than those of BMC/CTX.

In conclusion, the results of this pilot study indicate the beneficial effects of BM lysate in increasing the numbers of stem cells, while decreasing the numbers of myeloid cells with immunosuppressive phenotype. Further studies are ongoing to analyze the molecular components of these growth factors in BM lysate. This work represents an advance in biomedical science because this approach may shed light on a new strategy for the use of BM lysate or the secretome in combination with chemo-immunotherapy to optimize immune cell mobilization.

## Summary Table

### What is Known About This Subject?

• Leukopenia is one of the major side effects of myelosuppressive chemotherapy such as cyclophosphamide (CTX).
• Using CTX either alone or in combination with G-CSF for the mobilization of hematopoietic stem cells (HSCs).
• Mobilization of HSCs is often associated with the emergence of cells expressing the phenotype of MDSCs

### What This Paper Adds?

• BML/CTX can lower the expansion of myeloid cells in tumor-bearing mice
• BM lysate can mimic the mobilizing effects of G-CSF in a leukopenic microenvironment.
• The beneficial effects of BML to increase the numbers of stem cells with an immunosuppressive phenotype

## Concluding Statement

This work represents an advance in biomedical science because it may shed light on a new strategy for the use of BML in combination with chemo-immunotherapy to optimize immune cell mobilization.

## Data Availability

The original contributions presented in the study are included in the article/Supplementary Material, further inquiries can be directed to the corresponding author.

## Supplementary Material

Supplementary Figure S1 Representative data of Flow cytometry analysis: to illustrate the gating strategy of different myeloid cell subpopulations in PB and spleen. They include CD11b+Ly6G+ immature neutrophils. Ly6G+CD11b- mature neutrophils and Ly6G –CD11b + macrophage. Abbreviations: EAC, Ehrlisch Ascites carcinoma; CTX, cyclophosphamide; G-CSF, granulocyte-colony stimulating factor; BMC /PBS, bone marrow cells extracted from Naïve mice; BMC/CTX, bone marrow cells extracted from CTX-treated mice; BML/PBS, bone marrow lysate extracted from Naïve mice; BML/CTX, bone marrow lysate extracted from CTX-treated mice.

Supplementary Figure S2 The total number and the relative number of myeloid cell subpopulations of mature neutrophils in the PBL and spleen. **(A)** Total number of Ly6G+CD11b- in PBL **(B)** The percentage of Ly6G+CD11b- in PBL **(C)** Total number of Ly6G+CD11b- in the spleen **(D)** The percentage of Ly6G+CD11b- in the spleen. Columns represent the mean ± SE (*n* = 6). * Refers to *p* = 0.05, ** Refers to *p* ≤ 0.05, *** Refers to *p* < 0.001. Abbreviations: EAC, Ehrlisch Ascites carcinoma; CTX, cyclophosphamide; G-CSF, granulocyte-colony stimulating factor; BMC /PBS, bone marrow cells extracted from Naïve mice; BMC/CTX, bone marrow cells extracted from CTX-treated mice; BML/PBS, bone marrow lysate extracted from Naïve mice; BML/CTX, bone marrow lysate extracted from CTX-treated mice.
