# Supplementary figures and images for: Beneficial Modulatory Effects of Treatment With Bone Marrow Lysate on Hematopoietic Stem Cells and Myeloid Cells in Tumor-Bearing Mice
Source: Br J Biomed Sci. 2022 Jun 29;79:10328. doi: 10.3389/bjbs.2022.10328 (PMC9302549; doi:10.3389/bjbs.2022.10328)

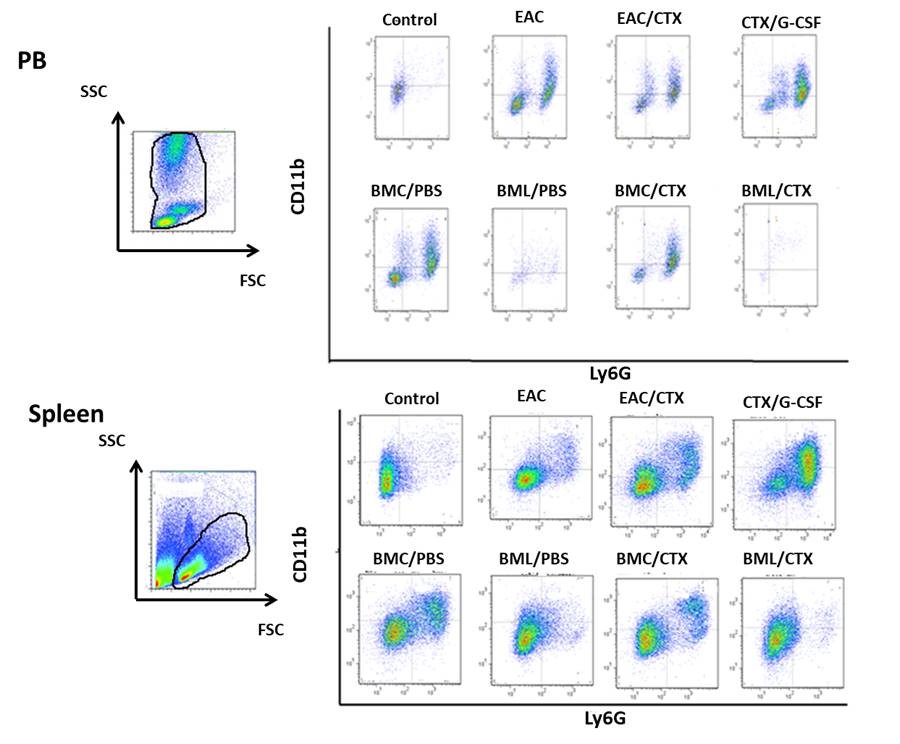

Supplement: Supplementary file 1 [file image1.jpeg]

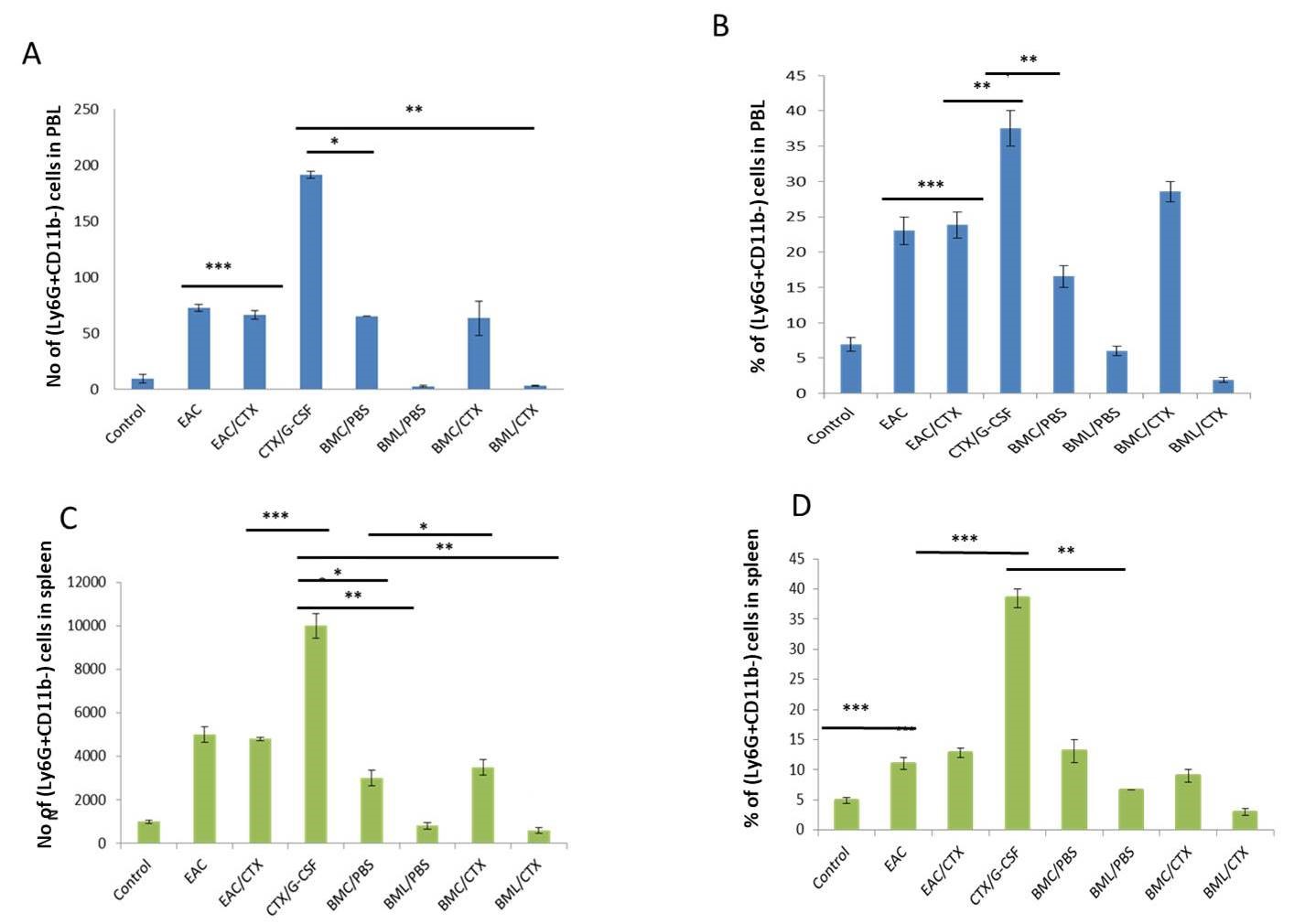

Supplement: Supplementary file 2 [file image2.jpeg]
